# A Unique Protein Adjuvant for Precision Immunotherapy to Prevent Recurrence of Surgically Resected Colorectal Cancer

**DOI:** 10.3390/cancers18061003

**Published:** 2026-03-20

**Authors:** Yasuhiro Suzuki, Rajesh Mani, B. Mark Evers

**Affiliations:** 1 Department of Microbiology, Immunology and Molecular Genetics, University of Kentucky College of Medicine, Lexington, KY 40536, USA; 2 Markey Cancer Center, University of Kentucky College of Medicine, Lexington, KY 40536, USA; 3 Department of Surgery, University of Kentucky College of Medicine, Lexington, KY 40536, USA

**Keywords:** immunotherapy, colorectal cancer, recurrence prevention, adjuvant, CD8^+^ T cells, cytotoxic T cells, IFN-γ, IL-18

## Abstract

Effectively activating protective CD8^+^ T cell immunity specifically against cancer antigens is an important pathway to prevent the growth of various types of cancers. A major obstacle in this approach is variations in cancer antigens among patients. A valuable material to overcome the antigen variation among cancer patients is the use of each individual’s own cancer cells for immunization. In colorectal cancer (CRC), approximately one-third of the patients who receive curative surgical resection have a recurrence of cancer. Therefore, the use of surgically resected CRC for immunotherapy to specifically activate the protective CD8^+^ T cells against their own cancer cells is a valuable approach to prevent the recurrence of cancer. However, since cancer-specific antigens are often not strongly immunogenic, a potent immunostimulant is required as an adjuvant for efficiently facilitating the activation of cancer-specific protective CD8^+^ T cells. We recently identified that a protein molecule, the amino-terminus region of the dense granule protein 6 (GRA6Nt) of *Toxoplasma gondii*, selectively activates innate expressions of IFN-γ and IL-18 and functions as a powerful adjuvant when used in immunization with nonreplicable (treated with mitomycin C or irradiated) MC38 CRC cells to potently activate the cytotoxic activity and IFN-γ production of CD8^+^ T cells against cancer cells. In addition, immunization using the GRA6Nt protein adjuvant effectively inhibits the growth of identical CRC cells after its challenge implantation, which mimics a recurrence of the surgically resected CRC used for the immunizations. In contrast to the two nucleotide- or deoxynucleotide-based Toll-like receptor agonists currently being used as adjuvants in cancer immunotherapy in clinical settings, GRA6Nt is a protein molecule. Thus, the rGRA6Nt protein adjuvant provides a new pathway in cancer immunotherapy to effectively activate the protective CD8^+^ T cells specific for the individual’s cancer cells to prevent the recurrence of surgically resected CRC in patients.

## Simple Summary

Colorectal cancer (CRC) is the second leading cause of death among cancers. Even after curative surgical resection of CRC, 30–35% of patients have a recurrence of the cancer. The cytotoxic activity and IFN-γ production of CD8^+^ T cells are critical for preventing the growth of CRC. Therefore, after the surgical resection of CRC, immunizing the patients with their own resected cancer cells to effectively activate the protective CD8^+^ T cells specifically against their own cancer cells is a valuable approach to prevent the recurrence of cancer. In this approach, the use of a potent adjuvant that efficiently facilitates the activation of protective CD8^+^ T cells in immunization is critical. In contrast to the two nucleotide- or deoxynucleotide-based adjuvants currently being used in cancer immunotherapy in clinical settings, we recently identified a novel protein molecule that functions as a powerful adjuvant to activate the cytotoxic activity and IFN-γ production of CD8^+^ T cells specifically against CRC cells used in immunizations. In addition, immunization using this novel protein adjuvant potently inhibits the growth of identical cancer cells after its challenge implantation, which mimics the recurrence of cancer. This review will first overview the importance of activating the cytotoxic activity and IFN-γ production of CD8^+^ T cells in the protective immunity against CRC and highlight the uniqueness and effectiveness of the novel protein adjuvant to efficiently activate the protective CD8^+^ T cell immunity against target CRC cells and prevent their growth in a murine model with MC38 CRC.

## 1. Introduction

Colorectal cancer (CRC) is the second leading cause of death among cancers. In addition, the frequency of recurrence of CRC after their curative surgical resection is 30–35% [1,2]. Although cancer immunotherapy using immune checkpoint blockade (ICB) and chimeric antigen receptor (CAR) T cells have made notable progress in cancer treatment [3,4,5,6], each of these new therapies still has significant disadvantages and limitations. Since the immune system utilizes powerful proinflammatory mechanisms to eliminate target materials including cancers, it needs to be finely tuned to prevent unwanted prolonged and/or overly stimulated inflammatory responses that could cause tissue damage. The immune checkpoint proteins are important downregulators of the immune system for this purpose. However, cancer cells often express these molecules to suppress the immune responses against them. ICB immunotherapy aims to block this inhibitory mechanism and enhance anti-cancer immunity [3,4,5,6]. However, because of the antigen non-specificity of this therapy, ICB often induces unwanted, overly stimulated inflammatory immune responses to antigens unrelated to the cancer, which cause potentially serious toxic side effects [7,8]. In addition, ICB has shown only limited efficacy for managing CRC [3,4,5,6].

Since CAR T cells express antigen receptors composed of the antigen-specific binding component of a monoclonal antibody [9], they recognize a single-target antigen determined by the specificity of the antigen-binding component of the antibody. Therefore, applying CAR T cells to treat CRC is challenging because of the notable variations in cancer antigens among individual patients. Thus, it is important to develop a new precision immunotherapy capable of potently activating the protective immunity to CRC specifically for each patient to prevent the recurrence of surgically resected tumors.

For overcoming antigen variation among cancer patients, a valuable approach is the use of each individual’s own cancer cells for immunization. However, since the immunogenicity of cancer-specific antigens is often not strong, we need to employ a potent adjuvant in immunization with each individual’s cancer cells to efficiently activate cancer-specific protective T cells. Cancer-specific CD8^+^ cytotoxic T cells are capable of killing cancer cells [10], and the presence of tumor-infiltrating CD8^+^ T cells is an indicator of the positive prognosis of cancer patients [11,12]. Since interferon-gamma (IFN-γ) is known to be critical for activating CD8^+^ cytotoxic T cells [13,14], the use of an adjuvant capable of potently activating IFN-γ production by innate immune cells such as macrophages will be a valuable approach for developing a precision immunotherapy using each individual’s own cancer to potently activate CD8^+^ cytotoxic T cells to the cancer cells specifically.

*Toxoplasma gondii* is an obligate intracellular protozoan parasite capable of powerfully activating IFN-γ-mediated [15,16,17] and CD8^+^ cytotoxic T cell-mediated protective immunity [18,19]. *T. gondii* establishes a chronic infection in various organs, especially in the brain [20], and both IFN-γ production and the cytotoxic activity of CD8^+^ T cells are required for controlling this pathogen in the brain [16,18,19,21]. Importantly, we recently identified a protein molecule, the amino-terminus region of dense granule protein 6 (GRA6Nt), of *T. gondii,* that activates the IFN-γ production of microglia, which are brain-resident macrophages [22]. Therefore, GRA6Nt appears to be an ideal molecule as an adjuvant for precision cancer immunotherapy utilizing cancer cells surgically resected from CRC patients to potently activate CD8^+^ cytotoxic T cells specifically to their own cancer cells. We recently examined the adjuvant activity of GRA6Nt of *T. gondii* using a murine model with MC38 CRC and discovered its powerful ability to potently activate both the cytotoxic activity and IFN-γ production of CD8^+^ T cells specifically against MC38 CRC cells specifically when used in the immunization of cancer cells. In addition, this immunization using the GRA6Nt protein adjuvant confers a powerful protection against a challenge implantation of the MC38 CRC cells as a model of the recurrence of surgically resected CRC [23,24]. In this review, we provide an overview on whole-cell cancer vaccines, the biology of adjuvants currently used in cancer therapy in clinical settings, and the uniqueness and efficiency of GRA6Nt as an adjuvant in immunotherapy utilizing whole cancer cells to powerfully activate IFN-γ production and the cytotoxic activity of CD8^+^ T cells specifically against cancer cells for protection against the growth of cancer cells identical to that used in immunotherapy.

## 2. Activation of Both Cytotoxic Activity and IFN-γ Production of Cancer-Specific CD8^+^ T Cells Is Important for Protective Immunity Against CRC

### 2.1. Importance of CD8^+^ Cytotoxic T Cells

Antigen-presenting cells (APCs), such as dendritic cells (DCs) and macrophages, uptake cancer antigens, process the antigen through the antigen cross-presentation pathway, and present the processed epitope peptides through the MHC class I molecules on their surfaces [25,26,27]. These APCs migrate to the secondary lymphoid organs through the CCR7 chemokine receptor expressed on their surface. Native CD8^+^ T cells in the lymphoid organs, which express T cell receptors (TCRs) specific to those cancer antigens, recognize those cancer antigens presented on the surface of the migrated APCs through their TCR and differentiate them into CD8^+^ cytotoxic effector T cells specific to those cancer antigens. Cytokines present during the T cell differentiation process also play a critical role in directing the differentiation of CD8^+^ T cells to cytotoxic effector T cells. These CD8^+^ cytotoxic T cells then go into systemic circulation and migrate to the site(s) where the cancer is growing and attack the cancer cells expressing the specific antigens that the CD8^+^ T cells recognize [25,26,27]. Once the CD8^+^ cytotoxic T cells recognize their target cancer cells, they secrete multiple cytotoxic molecules including perforin and granzyme B (GzmB) and kill the target cancer cells [25,26,27]. Cancer-specific CD8^+^ cytotoxic T cells are a key element of anti-cancer protective immunity. Consistently, the high-density presence of tumor-infiltrated CD8^+^ T cells is an indicator of a better prognosis and survival for CRC patients [28,29].

### 2.2. IFN-γ Production by CD8^+^ T Cells Is Also Important

Tumor-infiltrated CD8^+^ T cells are the major producer of IFN-γ in various types of cancers [11,30,31]. IFN-γ displays anti-tumor effects through multiple pathways. One of the protective effects of IFN-γ is to suppress spontaneous CRC development. Notably, when the IFN-γ or IFN-γ receptor is deficient, adenomatous polyposis coli (Apc)-mediated intestinal tumor development in *Apc*^Min/+^ mice significantly increases [32,33]. IFN-γ can also suppress CRC cell proliferation in vitro [32]. In addition, IFN-γ incudes the apoptosis [34] and ferroptosis [35,36] of cancer cells. IFN-γ can also increase the expression of MHC class I molecules on cancer cells [37,38], which can enhance the killing of cancer cells by CD8^+^ cytotoxic T cells.

Another important IFN-γ-mediated protective pathway is to amplify the recruitment and activation of anti-cancer CD8^+^ T cells into the cancer site. IFN-γ enhances the expressions of CXCL9 and CXCL10 chemokines of macrophages in the cancer microenvironment [39,40]. These chemokines recruit CD8^+^ T cells through the CXCR3 chemokine receptor expressed on the T cells and into the cancers to inhibit their growth [41,42,43]. IFN-γ also increases the phagocytic and tumor-killing activity of macrophages [44,45]. This cytokine enhances the nitric oxide (NO) production of macrophages through the upregulation of inducible NO synthase (NOS2), and NO provokes tumor cell death through apoptosis and/or necrosis [46,47]. NOS2 expression by macrophages also activates endothelial cells to induce their expression of vascular cell adhesion protein-1 (VCAM-1), which facilitates the recruitment of IFN-γ-producing T cells into the tumor tissue [48]. IFN-γ also induces the maturation and activation of DCs by upregulating the expression of MHC class I molecules and costimulatory molecules required for activating CD8^+^ T cells [49,50]. IFN-γ also induces a high expression of the CCR7 chemokine receptor on DCs for their migration into the lymphoid organs and activates their production of interleukin 12 (IL-12) and IL-18 for activating CD8^+^ T cells [49,50,51].

## 3. Clinical Trials in the Past for Immunotherapy Using Surgically Resected, Autologous CRC to Prevent Recurrence of Cancer

An important advantage in the use of surgically resected, autologous CRC for immunotherapy for patients is its potential to activate protective immunity against multiple antigens specific to their own cancers. For effectively activating those protective immune responses, it is critical to employ a potent adjuvant capable of facilitating the activation of protective CD8^+^ T cells specific to those cancer cells. *Mycobacterium bovis* bacillus Callmette Guerin (BCG) is currently used as an immunostimulant for the treatment of bladder cancer and metastatic melanoma in clinical settings [52,53]. An important effect of BCG is to induce the maturation of DCs [54,55], which are key antigen-presenting cells in the immune system. Thus, BCG most likely functions as an adjuvant to facilitate the activation of protective T cells against cancer-specific antigens through upregulating the antigen-presentation activity of DCs [56]. OncoVAX (Vaccinogen, Inc., Baltimore, MD, USA.) is a vaccine utilizing irradiated (non-tumorigenic) autologous cancer cells with a BCG adjuvant. Three clinical trials have reported on the use of OncoVAX to prevent the recurrence of surgically resected CRC [57,58,59]. One study with stage II CRC patients reported that OncoVAX increased the 5-year survival rates by 15.4% (87.6% in the treated group vs. 72.2% in the control group, *p* = 0.017) and 5-year disease-free survival rates by 15.9% (83.6% in the treated group vs. 67.7% in the control group, *p* = 0.006) [58]. Another study reported that the percentages of 5-year recurrence-free survival rates were 78.7% in the OncoVAX-treated group and 62.3% in the control group in stage II CRC patients (*p* = 0.009), but in stage III patients, the treatment did not prevent the recurrence of CRC and did not improve their disease-free survival rates [59]. In the third study, the 5-year overall survival rates were 85.5% in the treated group vs. 72.7% in the control group (*p* = 0.008) in stage II patients [57]. Although these clinical trials did not examine whether the OncoVAX treatment activated protective immunity specifically against the autologous cancer cells, those studies provided important evidence for the ability of immunotherapy using whole autologous CRC cells resected from patients to significantly reduce the recurrence of surgically resected tumors. These results also suggest that if we employ an adjuvant capable of activating IFN-γ production and the cytotoxic activity of CD8^+^ T cells specific against CRC cells used in immunotherapy more effectively than BCG does, we could open a door for efficiently preventing the recurrence of surgically resected CRC in a majority, if not all, of the patients.

## 4. Use of Chemical Adjuvants in Cancer Immunotherapy

Innate immune cells such as macrophages and DCs promptly detect pathogen-associated molecular patterns (PAMPs) during the early stages of microbial infections and become activated to initiate the protective immunity against pathogens. Toll-like receptors (TLRs) are a major host defense system that recognizes the PAMPs derived from bacteria, viruses, and parasites. Among the TLRs, TLR3 recognizes double-stranded RNA derived from viruses, and TLR9 recognizes DNA with unmethylated CpG of bacteria and herpesviruses. Currently, a TLR9 agonist, unmethylated CpG deoxynucleotides (CpG ODN) [60,61,62], and a TLR3 agonist, polyinosinic–polycytidylic acid [Poly(I:C)] [63,64], are used as adjuvants for cancer immunotherapy in clinical settings. The binding of these ligands to TLR3 or TLR9 activates the innate immune cells and induces their expression of type I IFN (IFN-α/β) and NF-κB-mediated proinflammatory cytokines such as IL-1β and TNF-α.

We are unable to find any articles reporting clinical trials of immunotherapy using CpG ODN or Poly(I:C) in combination with surgically resected, autologous CRC to prevent the recurrence of cancer. In murine models, there is one study that examined the protective effects of immunizations with cancer antigens plus CpG ODN adjuvants against a challenge implantation of MC38 CRC in mice [65]. In that study, mice received immunizations with co-assembled nanocomplexes of peptide neoantigen Adpgk, which is derived from MC38 CRC, plus a CpG ODN adjuvant three times with one-week intervals, followed by a subcutaneous challenge implantation of MC38 CRC cells (5 × 10^5^ cells) [65]. One in five mice in the immunized group survived for 60 days after the challenge implantation of CRC, whereas all control mice immunized with PBS died around 30 days after the challenge implantation of the cancer [65]. In this study, it is unclear whether the one mouse in the immunized group, which survived until 60 days after the tumor challenge, eventually died due to tumor growth at a later time point or if this mouse ultimately rejected the growth of the implanted tumor.

In the use of the Poly(I:C) adjuvant, we are unable to find any articles reporting the effects of immunization using this adjuvant against the challenge implantation of CRC. There is one article reporting that immunizations with the carcinoembryonic antigen (CEA) plus Poly(I:C) adjuvant can activate the cytotoxic activity of splenocytes against transduced MC38 CRC cells expressing CEA and significantly delay the growth of CEA-expressing MC38 CRC after its challenge implantation [66]. However, this immunization did not activate the cytotoxic activity of splenocytes against wild-type MC38 CRC cells [66]. Based on these observations, it is crucial to explore identifying a new adjuvant capable of more effectively activating both IFN-γ production and the cytotoxic activity of CD8^+^ T cells specifically against CRC than CpG ODN and Poly(I:C) do in immunotherapy utilizing whole CRC cells prepared from the surgically resected own tumors cells in patients for the prevention of the recurrence of cancer.

## 5. *T. gondii* Has a Unique Ability to Potently Activate Both Cytotoxic Activity and IFN-γ Production in CD8^+^ T Cell Immunity

*T. gondii* forms two different life cycle stages, the tachyzoite and the cyst, in mammalian hosts such as humans [67]. We originally identified the requirement of IFN-γ for controlling the proliferation of tachyzoites during the acute acquired stage of infection with *T. gondii* [15]. CD8^+^ T cells play a major role in the IFN-γ-mediated protective immunity against acute infection with this parasite, and CD4^+^ T cells also participate in the protective immunity [17,68]. Whereas the IFN-γ-mediated protective immunity effectively inhibits the proliferation of tachyzoites, the parasite transforms to the cysts and establishes a chronic infection, preferentially in the brain. This chronic infection can reactivate in immunocompromised individuals such as those with AIDS, neoplasms, and organ transplants, and develop potentially life-threatening toxoplasmic encephalitis (TE) [20]. IFN-γ is required for preventing the cerebral proliferation of tachyzoites and the development of TE [16,21,69]. The immune system also has the ability to attack the cyst stage of *T. gondii*. Notably, the anti-cyst immunity utilizes the perforin-dependent cytotoxic activity of CD8^+^ T cells [18,19], and IFN-γ production by CD8^+^ T cells is not required for this anti-cyst protective immunity [18]. Thus, *T. gondii* has the ability to potently activate both IFN-γ production and the cytotoxic activity of CD8^+^ T cells. Therefore, there is an excellent possibility that *T. gondii* has a molecule(s) that functions as an adjuvant to facilitate the potent activation of IFN-γ production and the cytotoxic activity of CD8^+^ T cells against this parasite.

The possibility of the presence of a powerful adjuvant molecule(s) in *T. gondii* for promoting the activation of protective CD8^+^ T cells is supported by the fact that *T. gondii* has potent activity to facilitate the maturation and an accumulation of DCs [70,71]. Our previous studies comparing the anti-cancer activity of the immune responses induced by *T. gondii* and those induced by BCG using subcutaneously implanted Lewis lung carcinoma (LLC) and EL4 lymphoma [72,73] revealed that the immune responses induced by *T. gondii* are much more effective than those induced by BCG to inhibit or prevent the growth of these cancers [72,73]. Notably, when these microorganisms were injected into the pre-implanted LLC tumor once at either 1, 3, or 5 days after cancer implantation, only the *T. gondii*-induced immune responses led to the regression or delay in growth of the tumors [73]. Therefore, it would be possible that *T. gondii* functions more efficiently as an adjuvant than BCG does in immunotherapy against CRC.

## 6. CD8^+^ Cytotoxic T Cells Activated by *T. gondii* Have the Ability to Penetrate into a Target of Large Mass for Its Elimination with Collaboration from Phagocytes

*T. gondii* cysts are formed within host cells, and the cysts can grow into sizes over 100 μm in diameter by containing hundreds to thousands of bradyzoites [74]. In the process of anti-cyst protective immunity, CD8^+^ cytotoxic T cells first attach on the surface of cyst-harboring cells as they do in the killing of their target cancer cells. However, in contrast to the process of killing cancer cells, in which the CD8^+^ cytotoxic T cells secrete cytotoxic molecules such as perforin and granzyme B to the target cancer cells to induce their death, CD8^+^ T cells penetrate into the cysts, which are much larger in size than the CD8^+^ T cells, as mentioned earlier, in a perforin-mediated mechanism [19]. The penetrated CD8^+^ T cells then secrete granzyme B into the cysts, and granular structures that are intensely positive for GzmB associate with bradyzoites within the cysts [19]. Their penetration results in morphological deterioration and destruction of the cysts and accumulation of microglia and macrophages into the cysts for the elimination of bradyzoites located within the cysts [19]. If CD8^+^ cytotoxic T cells penetrate solid tumors and secrete cytotoxic GzmB and possibly IFN-γ and induce the accumulation and activation of anti-cancer macrophages into the tumors, they could be very effective at preventing cancer growth and inducing their eradication. Since *T. gondii* potently activates the penetrating cytotoxic activity and IFN-γ production of CD8^+^ T cells, it is most likely that *T. gondii* has or secretes the molecule(s) that potently activate innate immune cells and facilitate the activation of the cytotoxic activity and IFN-γ production of CD8^+^ T cells. It is possible that such *T. gondii* molecule(s) can function as an effective adjuvant to facilitate the activation of cytotoxic activity and the IFN-γ production of CD8^+^ T cells against CRC when used in the immunization with cancer cells.

## 7. *T. gondii* GRA6Nt Protein Functions as Potent Adjuvant to Amplify Activation of Both Cytotoxic Activity and IFN-γ Production of CD8^+^ T Cells Specific to CRC for Preventing the Growth of Cancer

### 7.1. GRA6Nt Protein Selectively Activates Innate IFN-γ and IL-18 Expression

Cytokines produced by innate immune cells during the activation of naïve CD8^+^ T cells with their target antigen play crucial roles in determining the direction of their differentiation into effector T cells. IFN-γ is critical for activating CD8^+^ cytotoxic T cells [13,14], as mentioned earlier. IL-12, IL-15, and IL-18 also facilitate the activation of CD8^+^ T cells [75,76,77]. Therefore, the *T. gondii* molecule(s) that potently activates the innate production of IFN-γ in combination with IL-12, IL-15, and/or IL-18 will most likely function as an effective adjuvant to potently activate CD8^+^ cytotoxic T cells against target antigens such as CRC antigens.

**Figure 1:**
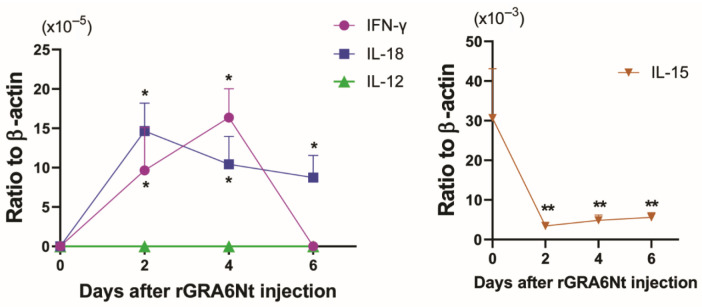
rGRA6Nt protein adjuvant selectively upregulates mRNA expression for IFN-γ and IL-18, but not for IL-12 and IL-15, in innate immune cells. The data show mRNA levels for these cytokines in the peritoneal innate immune cells of SCID mice deficient in T and B cells after an intraperitoneal injection of rGRA6Nt. * *p* < 0.05 and ** *p* < 0.01 when compared to day 0 (adapted from Figure 1 of reference [24]).

We recently identified that the GRA6Nt of *T. gondii* activates the IFN-γ production of microglia, the brain-resident macrophages [22]. Therefore, GRA6Nt could function as an effective adjuvant to activate CD8^+^ cytotoxic T cells against cancer antigens when used in the immunotherapy with cancer cells or cancer antigens. When SCID mice deficient in T and B cells are injected intraperitoneally with rGRA6Nt (40 μg), markedly increased levels of mRNA are detected only for IFN-γ and IL-18 in their peritoneal innate immune cells at 2 and 4 days after the injection of the rGRA6Nt adjuvant (Figure 1) [24]. In contrast, the mRNA levels for IL-12 and IL-15 in their peritoneal innate immune cells remain undetectable or rather decrease after the rGRA6Nt injection (Figure 1) [24]. Thus, the rGRA6Nt of *T. gondii* is able to selectively and promptly activate expressions of mRNA for IFN-γ and IL-18 in the innate immune cells.

The currently used CpG ODN and Poly I:C adjuvants in cancer immunotherapy in clinical settings are a TLR9 agonist [60,61,62] and a TLR3 agonist [63,64], respectively, and they activate the expression of type I IFN and NF-κB-mediated proinflammatory cytokines, as mentioned earlier in Section 4. Whereas type I IFN is known to have the activity to enhance CD8^+^ cytotoxic T cell activation [78,79], a study using peptide- or DNA-based vaccination against hepatitis B and HIV viruses demonstrated that mice deficient in the IFN-β or type I IFN receptor activate larger numbers of IFN-γ-producing CD8^+^ T cells than wild-type control mice [80], indicating that type I IFN downregulates the activation of IFN-γ production by CD8^+^ T cells during immunization. This point is further confirmed by the evidence that low-dose (500–1000 U/mouse) injections of IFN-α or IFN-β into wild-type mice downregulate the priming of IFN-γ-producing CD8^+^ T cells [80]. In addition, type I IFN facilitates the development of CD4^+^ T cells that produce immunosuppressive IL-10 in the vaccination [80]. Type I IFN also facilitates the development of IL-10-producing CD8^+^ T cells in a viral infection [81].

In contrast, IL-18-induced IFN-γ production by CD8^+^ T cells are much more resistant to the suppressive effects of IL-4 than IL-12- or IL-15-induced IFN-γ production by the T cells in a viral infection [82]. In addition, whereas IL-10 potently inhibits IFN-γ production of CD8^+^ T cells induced by IL-12 or IL-15, IL-10, in combination with IL-18, strongly enhances the IFN-γ production of CD8^+^ T cells in the infection [82]. Therefore, the selective upregulation of the innate production of IL-18 and IFN-γ appears to be ideal for overcoming an immunosuppressive microenvironment with IL-4 and IL-10, which cancer cells could induce in the tissue area where the immunization is provided, to potently activate the IFN-γ production of CD8^+^ T cells. The same could also be the case in the activation of the cytotoxic activity of CD8^+^ T cells. Therefore, it is possible that the GRA6Nt protein adjuvant capable of selectively activating IFN-γ and IL-18 expression in innate immune cells will provide a novel and effective pathway to activate anti-cancer protective immunity in the immunotherapy using whole cancer cells for conferring a protection against cancer cells, even if the cancer cells induce an immunosuppressive environment during immunization.

### 7.2. Immunizations with Nonreplicable CRC Cells in Combination with rGRA6Nt Protein Adjuvant Potently Activate Both the Cytotoxic Activity and IFN-γ Production of CD8^+^ T Cells Against Cancer Cells

**Figure 2:**
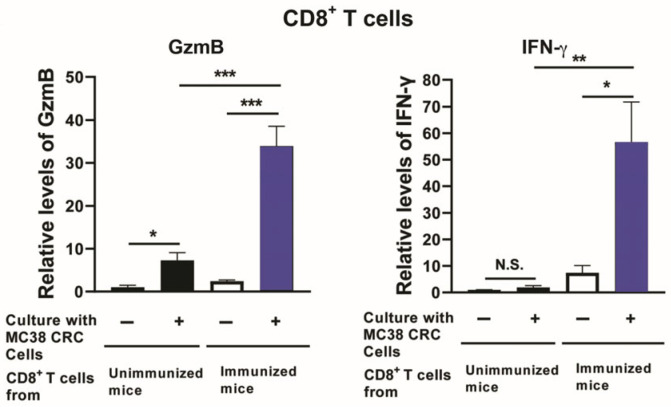
Immunizations with nonreplicable (treated with mitomycin C) MC38 CRC cells plus rGRA6Nt protein adjuvant are able to potently activate both the cytotoxic activity and IFN-γ production of CD8^+^ T cells against cancer cells. * *p* < 0.05, ** *p* < 0.01, and *** *p* < 0.001. N.S., not significant. (Modified from Figure 4 of reference [23]).

Activating the cytotoxic activity and IFN-γ production of CD8^+^ T cells specifically to CRC is crucial for preventing the growth of CRC, as mentioned earlier in Section 2. Thus, in the setting of CRC patients who received a curative surgical resection of their cancers, the use of their own resected CRC cells for an immunotherapy to potently activate the cytotoxic activity and IFN-γ production of CD8^+^ T cells specifically against their own cancer cells is an important approach to prevent the recurrence of their cancers. For determining the efficiency of the *T. gondii* rGRA6Nt protein as an adjuvant to facilitate the CD8^+^ T cells against CRC, immunizations with nonreplicable (treated with mitomycin C [MMC] or irradiated) MC38 CRC cells (1 × 10^6^ cells) plus the rGRA6Nt protein adjuvant (40 μg) are applied to mice twice, with a 4-week interval. CD8^+^ T cells purified from the spleens of mice at 2 weeks after the second immunization secrete large amounts of GzmB, a key mediator of the cytotoxic activity of CD8^+^ T cells, and IFN-γ in vitro only when their target MC38 CRC cells are present in the culture (Figure 2) [23]. This is a clear contrast to the CD8^+^ T cells from unimmunized mice, which secrete little or only small amounts only of GzmB and IFN-γ in response to MC38 CRC cells (Figure 2) [23]. Therefore, the rGRA6Nt protein of *T. gondii* functions as a powerful adjuvant when applied to immunization with nonreplicable CRC cells and induces the potent activation of both the cytotoxic activity and IFN-γ production of CD8^+^ T cells against cancer cells specifically.

### 7.3. Immunizations with Nonreplicable CRC Cells in Combination with rGRA6Nt Protein Adjuvant Confer a Potent Protection Against a Challenge Implantation with the Identical Cancer Cells

**Figure 3:**
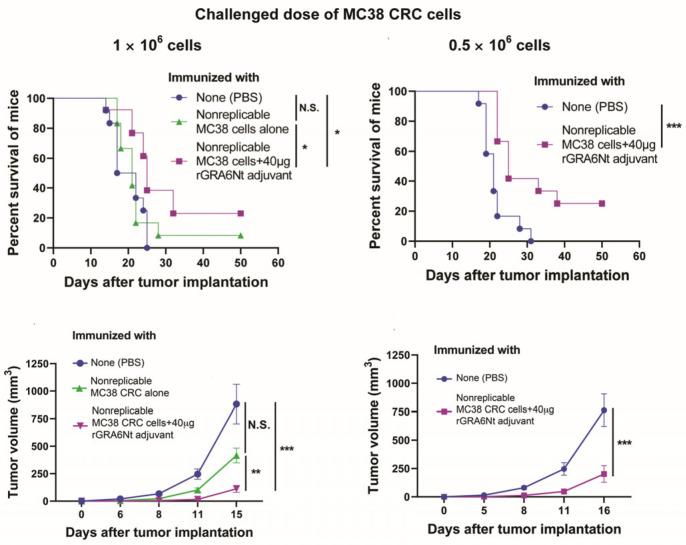
Immunizations with nonreplicable MC38 CRC cells plus rGRA6Nt protein adjuvant confer powerful protection against a challenge implantation of the identical cancer cells as a model of recurrence of surgically resected CRC. * *p* < 0.05, ** *p* < 0.01, and *** *p* < 0.001. N.S., not significant. (Modified from Figure 2 of reference [24]).

Applying a challenge implantation of MC38 CRC cells to mice immunized with nonreplicable MC38 CRC cells plus the rGRA6Nt protein adjuvant can be a model for examining the protective effects of immunizations to prevent the recurrence of CRC. After a challenge implantation of replication-capable MC38 CRC cells (1 and 0.5 × 10^6^ cells), the immunized mice significantly inhibit the growth of the implanted tumors when compared to unimmunized mice (*p* < 0.001, lower panels, Figure 3) [24]. Notably, 20 or 25% of immunized mice completely reject the growth of the implanted MC38 tumors after a challenge implantation of cancer cells, depending on the doses of the cancer cells used in the challenge implantation (upper panels in Figure 3) [24]. Overall, 22.2% (6/27) of immunized mice are able to reject the growth of the implanted MC38 tumors, whereas none (0/27) of the unimmunized mice are able to reject the implanted tumors (*p* < 0.05).

**Figure 4:**
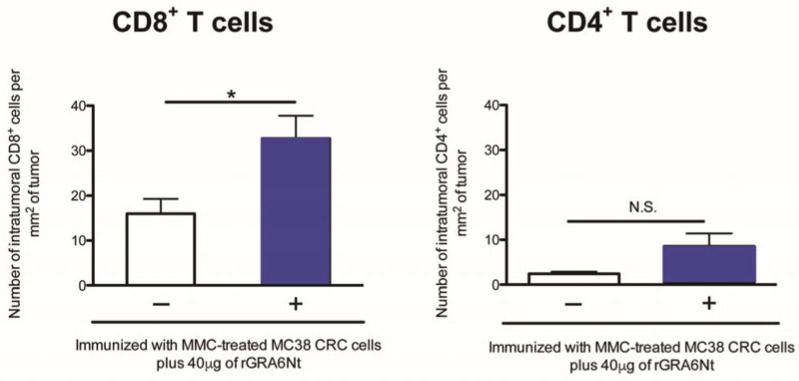
Immunizations with nonreplicable MC38 CRC cells plus rGRA6Nt adjuvant are able to increase the density of CD8^+^ T cells within MC38 tumors grown after its challenge implantation. * *p* < 0.05. N.S., not significant. (Modified from Figure 3 of reference [23]).

Notably, significantly greater numbers of CD8^+^ T cells, but not CD4^+^ T cells, are detectable within the tumors grown in the immunized mice than within those grown in unimmunized mice (Figure 4) (*p* < 0.05) [24]. Therefore, along with the potent cytotoxic activity and IFN-γ production of CD8^+^ T cells of the immunized mice against cancer cells, as shown in Figure 2, the protective effects of immunization using the rGRA6Nt protein adjuvant are most likely mediated by the cancer-specific CD8^+^ T cells. Importantly, the mice immunized with nonreplicable MC38CRC cells plus the rGRA6Nt protein adjuvant remain healthy and do not display any clinical signs of illness, suggesting that this immunization induces well-tuned protective CD8^+^ T cell responses and does not induce overly activated or prolonged immune responses that could cause harmful effects.

### 7.4. The Protective Effect of Immunizations with Nonreplicable CRC Cells Plus rGRA6Nt Protein Adjuvant Is Specific Against the Cancer Cells Used in Immunization

**Figure 5:**
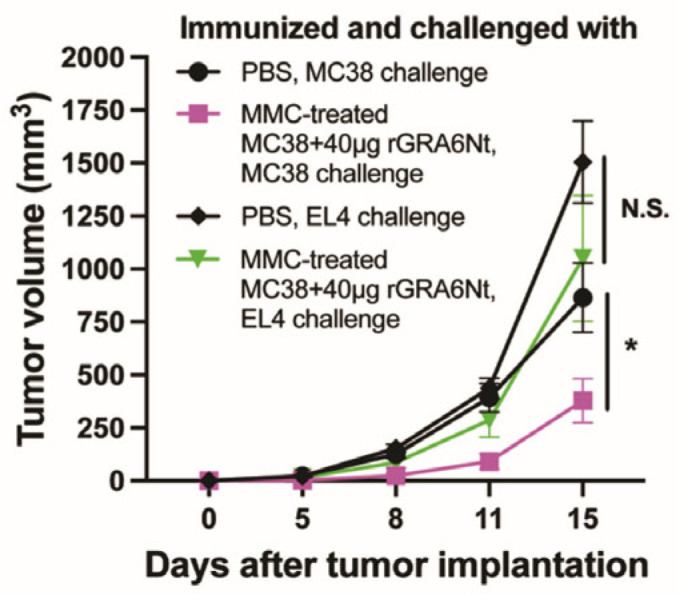
Immunizations with nonreplicable MC38 CRC cells plus rGRA6Nt protein adjuvant inhibit the growth of MC38 tumors but not EL4 lymphoma tumors after their challenge implantations. * *p* < 0.05. N.S., not significant. (Modified from Figure 5 of reference [23]).

Mice immunized with nonreplicable MC38 CRC cells plus the rGRA6Nt protein adjuvant are challenged with an implantation of MC38 CRC or EL4 lymphoma cells to determine the specificity of the protective effect of the immunization. Significant inhibition of the growth of implanted tumors is detected against MC38 CRC tumors only (*p* < 0.05) (Figure 5) [23]. Therefore, immunizations with nonreplicable CRC cells plus rGRA6Nt adjuvant confer a protection against the challenge implantation of cancer cells specifically to the CRC cells used in the immunization, supporting a potential use of the rGRA6Nt adjuvant for precision immunotherapy utilizing nonreplicable CRC cells prepared from surgically resected own tumors from each patient to potently activate the cancer cell-specific protective CD8^+^ T cells for preventing the recurrence of their CRC.

## 8. Conclusions

Since powerful proinflammatory responses are utilized by the immune system to eradicate foreign materials, including cancers, activating the protective immune responses specific to cancer antigens only is crucial to avoid toxic side effects that can be caused by overly stimulated immune responses to unrelated antigens in immunotherapy. For this purpose, immunotherapy using cancer cells obtained from surgically resected tumors from their own cancer for each individual patient is an ideal approach for activating the protective immunity specific to multiple antigens of their own cancer cells. Since the cytotoxic activity and IFN-γ production of CD8^+^ T cells are both key effector elements of anti-cancer protective immunity, there is an urgent need to develope an immunotherapy that can potently activate those protective activities of CD8^+^ T cells specific to the cancer cells of each individual patient. Since one-third of the CRC patients who receive curative surgical resection have a recurrence of cancer in current clinical settings [1,2], it is crucial to develop a precision immunotherapy using each individual’s surgically resected cancer cells to potently activate the cytotoxic activity and IFN-γ production of CD8^+^ T cells specific to their cancer cells in order to prevent the recurrence of their cancers.

**Figure 6:**
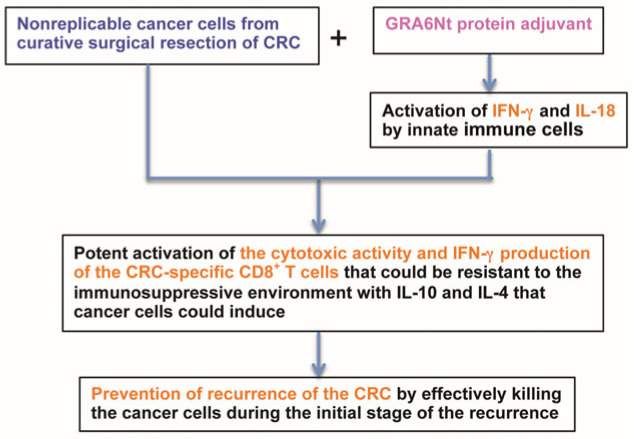
A schematic figure on prevention of recurrence of surgically resected CRC by immunotherapy utilizing nonreplicable, autologous cancer cells prepared from the resected tumors, in combination with novel GRA6Nt protein adjuvant in cancer patients.

Since cancer-specific antigens are not strongly immunogenic, an important element in the immunotherapy using surgically resected cancer cells is the use of a powerful adjuvant capable of efficiently facilitating the activation of cancer-specific CD8^+^ T cells. The rGRA6Nt protein of *T. gondii* is identified to have the capability of efficiently activating the innate production of IFN-γ and IL-18, both of which promote the activation of CD8^+^ T cells. Consistently applying the rGRA6Nt adjuvant in immunizations with nonreplicable (treated with mitomycin C or irradiated) MC38 CRC cells induces the potent activation of both of the two key effector functions, the cytotoxic activity and IFN-γ production, of CD8^+^ T cells specific to the cancer cells, and effectively inhibits the growth of tumors of the identical cancer cells after their challenge implantation as a model of the recurrence of the resected cancer. Notable evidence isthat more than one-fifth of the immunized mice eradicated the relatively large numbers (1 × 10^6^ or 5 × 10^5^ cells) of CRC cells after their challenge implantation. In clinical settings, the recurrence of surgically resected CRC tumors in cancer patients would most likely be initiated with very low numbers of the tumor cells, probably much lower than 1 × 10^6^ or 0.5 × 10^6^ cells used in the murine model described. Therefore, these observations confer a promising potential that, in CRC patients who receive the curative surgical resection of their tumors, immunizations with nonreplicable cancer cells of their own resected tumors with the powerful rGRA6Nt protein adjuvant provide a novel and valuable pathway to prevent the recurrence of tumors in those patients. Figure 6 shows a schematic diagram on the prevention of the recurrence of surgically resected CRC in cancer patients using immunotherapy utilizing nonreplicable, autologous cancer cells prepared from the resected tumors, in combination with the novel GRA6Nt protein adjuvant.

The described studies with relatively immunogenic MC38 CRC cells provide a critical foundation for expanding the potential of the novel rGRA6Nt protein adjuvant in studies with a less immunogenic CT26 CRC cell line and spontaneous CRC murine models, as well as studies with primary human CRC samples, which elucidate the efficacy of immunotherapy with the rGRA6Nt adjuvant to prevent the recurrence of surgically resected CRC.

## Data Availability

No new data were created or analyzed in this study.

## Abbreviations

The following abbreviations are used in this manuscript:

APCs	Antigen-presenting cells
CAR	Chimeric antigen receptor
CEA	Carcinoembryonic antigen
CpG ODN	Unmethylated CpG deoxynucleotides
CRC	Colorectal cancer
DCs	Dendritic cells
GRA6Nt	The amino-terminus region of dense granule protein 6
GzmB	Granzyme B
ICB	Immune checkpoint blockade
IFN-γ	Interferon-gamma
Interleukin	IL
MMC	Mitomycin C
NO	Nitric oxide
NOS2	Inducible nitric oxide synthase
Poly(I:C)	Polyinosinic-polycytidylic acid
TE	Toxoplasmic encephalitis
TLR	Toll-like receptor
TCR	T cell receptor
VCAM-1	Vascular cell adhesion protein-1

## References

[1] Young P.E., Womeldorph C.M., Johnson E.K., Maykel J.A., Brucher B., Stojadinovic A., Avital I., Nissan A., Steele S.R. (2014). Early detection of colorectal cancer recurrence in patients undergoing surgery with curative intent: Current status and challenges. J. Cancer.

[2] Scheer A., Auer R.A. (2009). Surveillance after curative resection of colorectal cancer. Clin. Colon Rectal Surg..

[3] Passardi A., Canale M., Valgiusti M., Ulivi P. (2017). Immune Checkpoints as a Target for Colorectal Cancer Treatment. Int. J. Mol. Sci..

[4] Arora S.P., Mahalingam D. (2018). Immunotherapy in colorectal cancer: For the select few or all?. J. Gastrointest. Oncol..

[5] Wang S., Hao J., Wang H., Fang Y., Tan L. (2018). Efficacy and safety of immune checkpoint inhibitors in non-small cell lung cancer. Oncoimmunology.

[6] Aguiar P.N., De Mello R.A., Barreto C.M.N., Perry L.A., Penny-Dimri J., Tadokoro H., Lopes G.L. (2017). Immune checkpoint inhibitors for advanced non-small cell lung cancer: Emerging sequencing for new treatment targets. ESMO Open.

[7] Postow M.A., Sidlow R., Hellmann M.D. (2018). Immune-Related Adverse Events Associated with Immune Checkpoint Blockade. N. Engl. J. Med..

[8] Wang D.Y., Salem J.E., Cohen J.V., Chandra S., Menzer C., Ye F., Zhao S., Das S., Beckermann K.E., Ha L. (2018). Fatal Toxic Effects Associated with Immune Checkpoint Inhibitors: A Systematic Review and Meta-analysis. JAMA Oncol..

[9] Jackson H.J., Rafiq S., Brentjens R.J. (2016). Driving CAR T-cells forward. Nat. Rev. Clin. Oncol..

[10] Martinez-Lostao L., Anel A., Pardo J. (2015). How Do Cytotoxic Lymphocytes Kill Cancer Cells?. Clin. Cancer Res..

[11] Farhood B., Najafi M., Mortezaee K. (2019). CD8^+^ cytotoxic T lymphocytes in cancer immunotherapy: A review. J. Cell. Physiol..

[12] Sato E., Olson S.H., Ahn J., Bundy B., Nishikawa H., Qian F., Jungbluth A.A., Frosina D., Gnjatic S., Ambrosone C. (2005). Intraepithelial CD8^+^ tumor-infiltrating lymphocytes and a high CD8^+^/regulatory T cell ratio are associated with favorable prognosis in ovarian cancer. Proc. Natl. Acad. Sci. USA.

[13] Roff S.R., Noon-Song E.N., Yamamoto J.K. (2014). The Significance of Interferon-gamma in HIV-1 Pathogenesis, Therapy, and Prophylaxis. Front. Immunol..

[14] Baird J.R., Byrne K.T., Lizotte P.H., Toraya-Brown S., Scarlett U.K., Alexander M.P., Sheen M.R., Fox B.A., Bzik D.J., Bosenberg M. (2013). Immune-mediated regression of established B16F10 melanoma by intratumoral injection of attenuated *Toxoplasma gondii* protects against rechallenge. J. Immunol..

[15] Suzuki Y., Orellana M.A., Schreiber R.D., Remington J.S. (1988). Interferon-gamma: The major mediator of resistance against *Toxoplasma gondii*. Science.

[16] Suzuki Y., Conley F.K., Remington J.S. (1989). Importance of endogenous IFN-gamma for prevention of toxoplasmic encephalitis in mice. J. Immunol..

[17] Gazzinelli R.T., Hakim F.T., Hieny S., Shearer G.M., Sher A. (1991). Synergistic role of CD4^+^ and CD8^+^ T lymphocytes in IFN-gamma production and protective immunity induced by an attenuated *Toxoplasma gondii* vaccine. J. Immunol..

[18] Suzuki Y., Wang X., Jortner B.S., Payne L., Ni Y., Michie S.A., Xu B., Kudo T., Perkins S. (2010). Removal of *Toxoplasma gondii* cysts from the brain by perforin-mediated activity of CD8^+^ T cells. Am. J. Pathol..

[19] Tiwari A., Hannah R., Lutshumba J., Ochiai E., Weiss L.M., Suzuki Y. (2019). Penetration of CD8^+^ cytotoxic T cells into large target, tissue cysts of *Toxoplasma gondii*, leads to its elimination. Am. J. Pathol..

[20] Montoya J.G., Liesenfeld O. (2004). Toxoplasmosis. Lancet.

[21] Gazzinelli R., Xu Y., Hieny S., Cheever A., Sher A. (1992). Simultaneous depletion of CD4^+^ and CD8^+^ T lymphocytes is required to reactivate chronic infection with *Toxoplasma gondii*. J. Immunol..

[22] Sa Q., Mercier C., Cesbron-Delauw M.F., Suzuki Y. (2020). The amino-terminal region of dense granule protein 6 of *Toxoplasma gondii* stimulates IFN-gamma production by microglia. Microbes Infect..

[23] Mani R., Martin C.G., Balu K.E., Wang Q., Rychahou P., Izumi T., Evers B.M., Suzuki Y. (2024). A novel protozoa parasite-derived protein adjuvant is effective in immunization with cancer cells to activate the cancer-specific protective immunity and inhibit the cancer growth in a murine model of colorectal cancer. Cells.

[24] Mani R., Evers B.M., Suzuki Y. (2025). Novel Protein Adjuvant Activating Innate IFN-gamma and IL-18 Expression and Inducing Rejection of Implanted Colorectal Cancer Following Immunotherapy Using This Adjuvant in Mice. Med. Res. Arch..

[25] Lin Y., Song Y., Zhang Y., Li X., Kan L., Han S. (2025). New insights on anti-tumor immunity of CD8^+^ T cells: Cancer stem cells, tumor immune microenvironment and immunotherapy. J. Transl. Med..

[26] Chen Y., Yu D., Qian H., Shi Y., Tao Z. (2024). CD8^+^ T cell-based cancer immunotherapy. J. Transl. Med..

[27] Jhunjhunwala S., Hammer C., Delamarre L. (2021). Antigen presentation in cancer: Insights into tumour immunogenicity and immune evasion. Nat. Rev. Cancer.

[28] Lalos A., Tulek A., Tosti N., Mechera R., Wilhelm A., Soysal S., Daester S., Kancherla V., Weixler B., Spagnoli G.C. (2021). Prognostic significance of CD8^+^ T-cells density in stage III colorectal cancer depends on SDF-1 expression. Sci. Rep..

[29] Wankhede D., Halama N., Kloor M., Edelmann D., Brenner H., Hoffmeister M. (2025). Prognostic value of CD8^+^ T cells at the invasive margin is comparable to the immune score in nonmetastatic colorectal cancer: A Prospective multicentric cohort study. Clin. Cancer Res..

[30] Komita H., Homma S., Saotome H., Zeniya M., Ohno T., Toda G. (2006). Interferon-gamma produced by interleukin-12-activated tumor infiltrating CD8^+^T cells directly induces apoptosis of mouse hepatocellular carcinoma. J. Hepatol..

[31] de Araujo-Souza P.S., Hanschke S.C.H., Nardy A., Secca C., Oliveira-Vieira B., Silva K.L., Soares-Lima S.C., Viola J.P.B. (2020). Differential interferon-gamma production by naive and memory-like CD8 T cells. J. Leucoc. Biol..

[32] Wang L., Wang Y., Song Z., Chu J., Qu X. (2015). Deficiency of interferon-gamma or its receptor promotes colorectal cancer development. J. Interferon Cytokine Res..

[33] Zhang C., Hou D., Wei H., Zhao M., Yang L., Liu Q., Zhang X., Gong Y., Shao C. (2016). Lack of interferon-gamma receptor results in a microenvironment favorable for intestinal tumorigenesis. Oncotarget.

[34] Kuang Q.X., Huang Y.Q., Ruan Y.Q., Lai H.Z., Long J., Yan C.Y., Lei H.R., Guo D.L., Deng Y., You F.M. (2024). New benzophenone analogs from Nigrospora sphaerica and their inhibitory activity against PD-1/PD-L1 interactions. Bioorg. Chem..

[35] Wang W., Green M., Choi J.E., Gijon M., Kennedy P.D., Johnson J.K., Liao P., Lang X., Kryczek I., Sell A. (2019). CD8^+^ T cells regulate tumour ferroptosis during cancer immunotherapy. Nature.

[36] Haoyue W., Kexiang S., Shan T.W., Jiamin G., Luyun Y., Junkai W., Wanli D. (2025). Icariin promoted ferroptosis by activating mitochondrial dysfunction to inhibit colorectal cancer and synergistically enhanced the efficacy of PD-1 inhibitors. Phytomedicine.

[37] Angelicola S., Ruzzi F., Landuzzi L., Scalambra L., Gelsomino F., Ardizzoni A., Nanni P., Lollini P.L., Palladini A. (2021). IFN-gamma and CD38 in hyperprogressive cancer development. Cancers.

[38] Corulli L.R., Cecil D.L., Gad E., Koehnlein M., Coveler A.L., Childs J.S., Lubet R.A., Disis M.L. (2021). Multi-epitope-based vaccines for colon cancer treatment and prevention. Front. Immunol..

[39] Bergamaschi C., Pandit H., Nagy B.A., Stellas D., Jensen S.M., Bear J., Cam M., Valentin A., Fox B.A., Felber B.K. (2020). Heterodimeric IL-15 delays tumor growth and promotes intratumoral CTL and dendritic cell accumulation by a cytokine network involving XCL1, IFN-gamma, CXCL9 and CXCL10. J. Immunother. Cancer.

[40] Liu L., Gao J., Xing X., Jiang M., Liu Q., Wang S., Luo Y. (2022). Cyclin G2 in macrophages triggers CTL-mediated antitumor immunity and antiangiogenesis via interferon-gamma. J. Exp. Clin. Cancer Res..

[41] Chheda Z.S., Sharma R.K., Jala V.R., Luster A.D., Haribabu B. (2016). Chemoattractant receptors BLT1 and CXCR3 regulate antitumor immunity by facilitating CD8^+^ T cell migration into tumors. J. Immunol..

[42] Yang X., Chu Y., Wang Y., Zhang R., Xiong S. (2006). Targeted in vivo expression of IFN-gamma-inducible protein 10 induces specific antitumor activity. J. Leukoc. Biol..

[43] Gorbachev A.V., Kobayashi H., Kudo D., Tannenbaum C.S., Finke J.H., Shu S., Farber J.M., Fairchild R.L. (2007). CXC chemokine ligand 9/monokine induced by IFN-gamma production by tumor cells is critical for T cell-mediated suppression of cutaneous tumors. J. Immunol..

[44] Celada A., Gray P.W., Rinderknecht E., Schreiber R.D. (1984). Evidence for a gamma-interferon receptor that regulates macrophage tumoricidal activity. J. Exp. Med..

[45] Castro F., Cardoso A.P., Goncalves R.M., Serre K., Oliveira M.J. (2018). Interferon-gamma at the crossroads of tumor immune surveillance or evasion. Front. Immunol..

[46] Brune B., Courtial N., Dehne N., Syed S.N., Weigert A. (2017). Macrophage NOS2 in tumor leukocytes. Antioxid. Redox Signal..

[47] Muller E., Speth M., Christopoulos P.F., Lunde A., Avdagic A., Oynebraten I., Corthay A. (2018). Both type I and type II interferons can activate antitumor M1 macrophages when combined with TLR stimulation. Front. Immunol..

[48] Paul S., Chhatar S., Mishra A., Lal G. (2019). Natural killer T cell activation increases iNOS^+^CD206^-^ M1 macrophage and controls the growth of solid tumor. J. Immunother. Cancer.

[49] Pan J., Zhang M., Wang J., Wang Q., Xia D., Sun W., Zhang L., Yu H., Liu Y., Cao X. (2004). Interferon-gamma is an autocrine mediator for dendritic cell maturation. Immunol. Lett..

[50] Fang P., Li X., Dai J., Cole L., Camacho J.A., Zhang Y., Ji Y., Wang J., Yang X.F., Wang H. (2018). Immune cell subset differentiation and tissue inflammation. J. Hematol. Oncol..

[51] Iwai Y., Hemmi H., Mizenina O., Kuroda S., Suda K., Steinman R.M. (2008). An IFN-gamma-IL-18 signaling loop accelerates memory CD8^+^ T cell proliferation. PLoS ONE.

[52] Fuge O., Vasdev N., Allchorne P., Green J.S. (2015). Immunotherapy for bladder cancer. Res. Rep. Urol..

[53] Kidner T.B., Morton D.L., Lee D.J., Hoban M., Foshag L.J., Turner R.R., Faries M.B. (2012). Combined intralesional Bacille Calmette-Guerin (BCG) and topical imiquimod for in-transit melanoma. J. Immunother..

[54] Kim K.D., Lee H.G., Kim J.K., Park S.N., Choe I.S., Choe Y.K., Kim S.J., Lee E., Lim J.S. (1999). Enhanced antigen-presenting activity and tumour necrosis factor-alpha-independent activation of dendritic cells following treatment with *Mycobacterium bovis* bacillus Calmette-Guerin. Immunology.

[55] Thurnher M., Ramoner R., Gastl G., Radmayr C., Bock G., Herold M., Klocker H., Bartsch G. (1997). Bacillus Calmette-Guerin mycobacteria stimulate human blood dendritic cells. Int. J. Cancer.

[56] Antonelli A.C., Binyamin A., Hohl T.M., Glickman M.S., Redelman-Sidi G. (2020). Bacterial immunotherapy for cancer induces CD4-dependent tumor-specific immunity through tumor-intrinsic interferon-gamma signaling. Proc. Natl. Acad. Sci. USA.

[57] Hanna M.G. (2012). Immunotherapy with autologous tumor cell vaccines for treatment of occult disease in early stage colon cancer. Hum. Vaccines Immunother..

[58] Hanna M.G., Hoover H.C., Vermorken J.B., Harris J.E., Pinedo H.M. (2001). Adjuvant active specific immunotherapy of stage II and stage III colon cancer with an autologous tumor cell vaccine: First randomized phase III trials show promise. Vaccine.

[59] Uyl-de Groot C.A., Vermorken J.B., Hanna M.G., Verboom P., Groot M.T., Bonsel G.J., Meijer C.J., Pinedo H.M. (2005). Immunotherapy with autologous tumor cell-BCG vaccine in patients with colon cancer: A prospective study of medical and economic benefits. Vaccine.

[60] Krieg A.M. (2007). Development of TLR9 agonists for cancer therapy. J. Clin. Investig..

[61] Carpentier A., Laigle-Donadey F., Zohar S., Capelle L., Behin A., Tibi A., Martin-Duverneuil N., Sanson M., Lacomblez L., Taillibert S. (2006). Phase 1 trial of a CpG oligodeoxynucleotide for patients with recurrent glioblastoma. Neuro Oncol..

[62] Muraoka D., Kato T., Wang L., Maeda Y., Noguchi T., Harada N., Takeda K., Yagita H., Guillaume P., Luescher I. (2010). Peptide vaccine induces enhanced tumor growth associated with apoptosis induction in CD8^+^ T cells. J. Immunol..

[63] Sultan H., Wu J., Fesenkova V.I., Fan A.E., Addis D., Salazar A.M., Celis E. (2020). Poly-IC enhances the effectiveness of cancer immunotherapy by promoting T cell tumor infiltration. J. Immunother. Cancer.

[64] De Waele J., Verhezen T., van der Heijden S., Berneman Z.N., Peeters M., Lardon F., Wouters A., Smits E. (2021). A systematic review on poly(I:C) and poly-ICLC in glioblastoma: Adjuvants coordinating the unlocking of immunotherapy. J. Exp. Clin. Cancer Res..

[65] Liang Z., Cui X., Yang L., Hu Q., Li D., Zhang X., Han L., Shi S., Shen Y., Zhao W. (2021). Co-assembled nanocomplexes of peptide neoantigen Adpgk and Toll-like receptor 9 agonist CpG ODN for efficient colorectal cancer immunotherapy. Int. J. Pharm..

[66] Park J.S., Kim H.S., Park H.M., Kim C.H., Kim T.G. (2011). Efficient induction of anti-tumor immunity by a TAT-CEA fusion protein vaccine with poly(I:C) in a murine colorectal tumor model. Vaccine.

[67] Dubey J.P., Baron S. (1996). *Toxoplasma gondii*. Medical Microbiology.

[68] Suzuki Y., Remington J.S. (1988). Dual regulation of resistance against *Toxoplasma gondii* infection by Lyt-2^+^ and Lyt-1^+^, L3T4^+^ T cells in mice. J. Immunol..

[69] Wang X., Kang H., Kikuchi T., Suzuki Y. (2004). Gamma interferon production, but not perforin-mediated cytolytic activity, of T cells is required for prevention of toxoplasmic encephalitis in BALB/c mice genetically resistant to the disease. Infect. Immun..

[70] Reis e Sousa C., Hieny S., Scharton-Kersten T., Jankovic D., Charest H., Germain R.N., Sher A. (1997). In vivo microbial stimulation induces rapid CD40 ligand-independent production of interleukin 12 by dendritic cells and their redistribution to T cell areas. J. Exp. Med..

[71] Sher A., Hieny S., Charest H., Scharton-Kersten T., Collazo C., Germain R.N., Reis e Sousa C. (1998). The role of dendritic cells in the initiation of host resistance to *Toxoplasma gondii*. Adv. Exp. Med. Biol..

[72] Suzuki Y., Muto M., Kobayashi A. (1986). Antitumor effect of formalin-fixed *Toxoplasma gondii* organisms on EL4 lymphoma in *Toxoplasma*-infected mice. J. Biol. Response Modif..

[73] Suzuki Y., Kobayashi A. (1985). Antitumor effect of intralesional injection with formalin-fixed *Toxoplasma gondii* organisms on Lewis lung carcinoma in *Toxoplasma*-infected mice. Cancer Lett..

[74] Lutshumba J., Ochiai E., Sa Q., Anand N., Suzuki Y. (2020). Selective upregulation of transcripts for six molecules related to T cell costimulation and phagocyte recruitment and activation among 734 immunity-related genes in the brain during perforin-dependent, CD8^+^ T cell-mediated elimination of *Toxoplasma gondii* cysts. mSystems.

[75] Henry C.J., Ornelles D.A., Mitchell L.M., Brzoza-Lewis K.L., Hiltbold E.M. (2008). IL-12 produced by dendritic cells augments CD8^+^ T cell activation through the production of the chemokines CCL1 and CCL17. J. Immunol..

[76] Khan I.A., Kasper L.H. (1996). IL-15 augments CD8+ T cell-mediated immunity against *Toxoplasma gondii* infection in mice. J. Immunol..

[77] Okamoto I., Kohno K., Tanimoto T., Ikegami H., Kurimoto M. (1999). Development of CD8^+^ effector T cells is differentially regulated by IL-18 and IL-12. J. Immunol..

[78] Fuertes M.B. (2011). Host type I IFN signals are required for antitumor CD8^+^ T cell responses through CD8a dendritic cells. J. Exp. Med..

[79] Busselaar J., Sijbranda M., Borst J. (2024). The importance of type I interferon in orchestrating the cytotoxic T-cell response to cancer. Immunol. Lett..

[80] Dikopoulos N., Bertoletti A., Kroger A., Hauser H., Schirmbeck R., Reimann J. (2005). Type I IFN negatively regulates CD8^+^ T cell responses through IL-10-producing CD4^+^ T regulatory 1 cells. J. Immunol..

[81] Jiang L., Yao S., Huang S., Wright J., Braciale T.J., Sun J. (2016). Type I IFN signaling facilitates the development of IL-10-producing effector CD8^+^ T cells during murine influenza virus infection. Eur. J. Immunol..

[82] Freeman B.E., Hammarlund E., Raue H.P., Slifka M.K. (2012). Regulation of innate CD8^+^ T-cell activation mediated by cytokines. Proc. Natl. Acad. Sci. USA.

